# Supplementary material for: Intrinsic Subtypes and Androgen Receptor Gene Expression in Primary Breast Cancer. A Meta-Analysis
Source: Biology (Basel). 2021 Aug 27;10(9):834. doi: 10.3390/biology10090834 (PMC8466727; doi:10.3390/biology10090834)
Supplement: Supplementary file 1 [file biology-10-00834-s001.zip › Cruz-Tapias_Supp Material.pdf]

Supplementary Materials: Intrinsic subtypes and Androgen Receptor gene expression in primary breast cancer. A meta-analysis

Paola Cruz-Tapias, Wilson Rubiano, Milena Rondón-Lagos, Victoria-Eugenia Villegas, Nelson Rangel

1A)

Study	Std diff in means	Lower limit	Upper limit	p-Value	Weight
GSE80999	0.794	0.471	1.118	<0.001	4.01%
GSE76727	-1.216	-2.610	0.178	0.087	1.44%
GSE49481	0.902	0.524	1.280	<0.001	3.87%
GSE60785	1.467	0.359	2.574	0.009	1.92%
GSE55639	0.000	-0.956	0.956	1.000	2.25%
GSE42568	0.000	-0.649	0.649	1.000	3.07%
GSE25307	-0.231	-0.555	0.093	0.162	4.01%
GSE31364	1.102	0.321	1.884	0.006	2.69%
GSE20711	0.677	0.076	1.278	0.027	3.21%
GSE31863	0.000	-0.436	0.436	1.000	3.71%
GSE24185	0.333	-0.257	0.924	0.269	3.24%
GSE22219	0.712	0.307	1.117	0.001	3.79%
GSE20194	0.332	-0.236	0.899	0.252	3.31%
GSE8757	-0.085	-0.469	0.299	0.664	3.85%
GSE7390	0.645	0.219	1.071	0.003	3.73%
GSE86166	0.608	0.243	0.973	0.001	3.90%
GSE59246	0.000	-0.606	0.606	1.000	3.20%
GSE78958	1.100	0.828	1.372	<0.001	4.13%
GSE61304	0.405	-0.532	1.343	0.397	2.29%
GSE43365	0.000	-0.538	0.538	1.000	3.40%
GSE45255	0.014	-0.518	0.546	0.959	3.42%
GSE10893	-0.786	-1.273	-0.299	0.002	3.56%
GSE16987	0.537	-0.041	1.115	0.069	3.28%
GSE26639	0.959	0.410	1.507	0.001	3.37%
GSE20271	0.744	0.177	1.311	0.010	3.31%
GSE20624	-0.771	-1.418	-0.125	0.019	3.08%
GSE21653	1.176	0.814	1.539	<0.001	3.91%
GSE11121	0.746	0.237	1.255	0.004	3.49%
GSE19615	0.928	0.431	1.424	<0.001	3.53%
GSE30682	0.772	0.450	1.095	<0.001	4.01%
**Total (95% CI)**	**0.427**	**0.223**	**0.630**	**< 0.001**	**100%**

Heterogeneity: Q-value = 150.97; df = 29 ($p < 0.001$); $I^2 = 80.79\%$

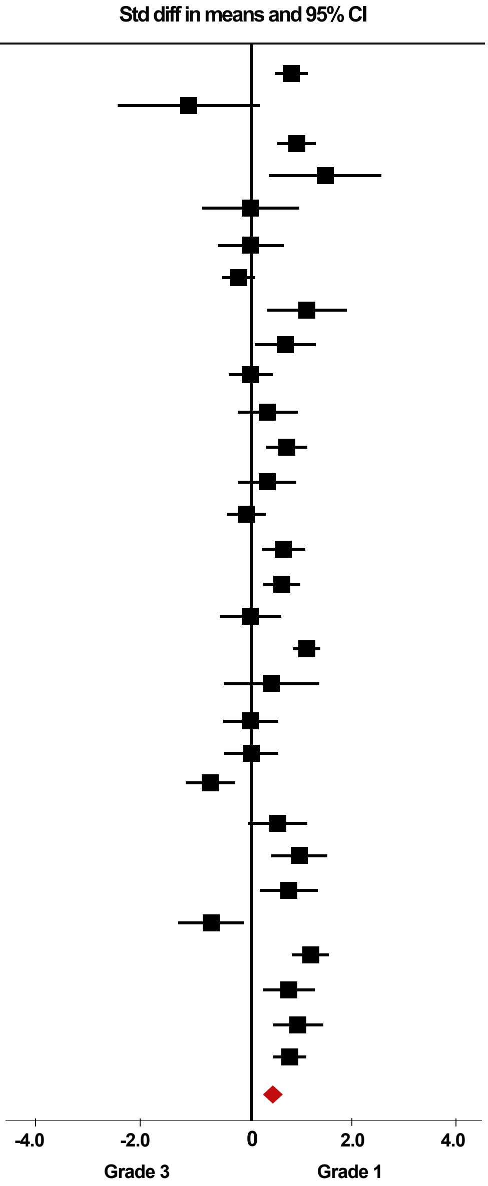

Supplementary Figure S1. *Cont.*

1B)

Study	Std diff in means	Lower limit	Upper limit	p-Value	Weight
GSE88847	1.142	-0.067	2.350	0.064	0.59%
GSE80999	0.579	0.350	0.809	< 0.001	3.80%
GSE76727	0.000	-0.733	0.733	1.000	1.32%
GSE49481	0.652	0.340	0.965	< 0.001	3.24%
GSE59590	0.332	0.019	0.645	0.037	3.24%
GSE60785	0.932	0.275	1.589	0.005	1.54%
GSE55639	0.000	-0.980	0.980	1.000	0.84%
GSE42568	0.327	-0.086	0.740	0.121	2.61%
GSE25307	0.000	-0.222	0.222	1.000	3.85%
GSE31364	0.620	0.026	1.214	0.041	1.76%
GSE20711	0.000	-0.907	0.907	1.000	0.96%
GSE31863	0.000	-0.437	0.437	1.000	2.47%
GSE24185	0.150	-0.276	0.576	0.491	2.54%
GSE22219	0.467	0.139	0.796	0.005	3.13%
GSE20194	0.410	0.157	0.663	0.001	3.64%
GSE10810	0.548	-0.345	1.440	0.229	0.98%
GSE8757	0.000	-0.347	0.347	1.000	3.01%
GSE7390	0.590	0.279	0.901	< 0.001	3.25%
GSE4056	0.550	0.076	1.023	0.023	2.29%
GSE86166	0.675	0.448	0.903	< 0.001	3.82%
GSE59246	0.241	-0.212	0.694	0.297	2.39%
GSE78958	0.837	0.613	1.061	< 0.001	3.84%
GSE61304	0.521	-0.073	1.116	0.086	1.76%
GSE29044	0.452	-0.059	0.962	0.083	2.11%
GSE43365	0.000	-0.422	0.422	1.000	2.56%
GSE45255	0.547	0.178	0.916	0.004	2.87%
GSE10893	0.000	-0.309	0.309	1.000	3.26%
GSE16987	0.380	0.039	0.721	0.029	3.05%
GSE22597	0.520	-0.032	1.072	0.065	1.93%
GSE26639	0.572	0.287	0.857	< 0.001	3.42%
GSE26304	0.539	0.128	0.951	0.010	2.62%
GSE20271	0.027	-0.314	0.368	0.878	3.05%
GSE20624	-0.114	-0.494	0.266	0.557	2.81%
GSE21653	0.776	0.494	1.057	< 0.001	3.45%
GSE11121	0.859	0.477	1.242	< 0.001	2.79%
GSE1561	0.766	0.139	1.394	0.017	1.64%
GSE17907	-0.029	-0.734	0.676	0.936	1.40%
GSE19615	0.309	-0.137	0.755	0.175	2.43%
GSE30682	0.587	0.349	0.825	< 0.001	3.75%
**Total (95% CI)**	**0.408**	**0.309**	**0.507**	**< 0.001**	**100%**

Heterogeneity: Q-value = 97.83; df = 38 ($p < 0.001$); I^2 = 61.15%

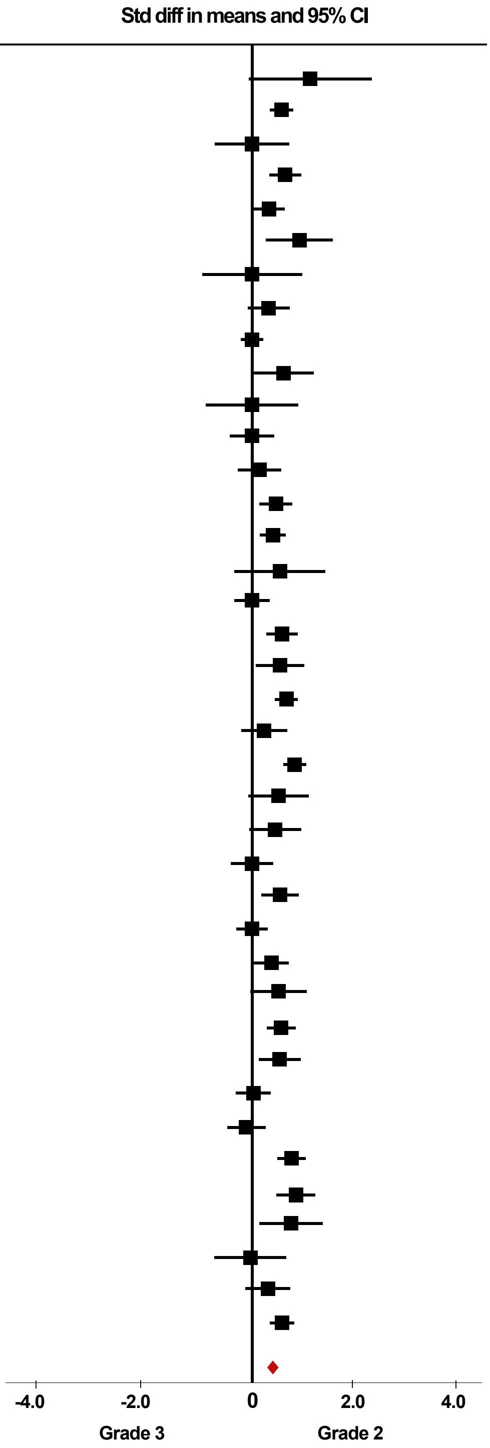

Supplementary Figure S1. *AR* mRNA expression is significantly increased in BC patients with low histological grade. Results from the primary analysis. Forest plots of SMD comparing *AR* mRNA levels in BC patients with histological grade 3 Vs BC patients with histological grade 1 **(A)**, and grade 2 **(B)**. SMD for each dataset are represented by the squares, and the horizontal line crossing the square represents the 95% CI. The red diamonds represent the estimated overall effect.

2A)

Study	Std diff in means	Lower limit	Upper limit	p-Value	Weight
GSE80999	1.158	0.875	1.441	< 0.001	2.21%
GSE89116	-0.322	-1.177	0.533	0.461	1.32%
GSE76360	-0.790	-1.369	-0.210	0.008	1.74%
GSE49481	1.017	0.709	1.325	< 0.001	2.17%
GSE59590	0.855	0.511	1.199	< 0.001	2.12%
GSE65194	1.205	0.830	1.580	< 0.001	2.08%
GSE60785	1.177	0.501	1.853	0.001	1.59%
GSE42568	0.762	0.336	1.188	< 0.001	2.00%
GSE37126	0.977	0.298	1.656	0.005	1.58%
GSE41998	1.502	1.231	1.773	< 0.001	2.22%
GSE25307	-0.124	-0.301	0.053	0.169	2.32%
GSE31364	0.507	-0.130	1.145	0.119	1.65%
GSE20711	0.580	0.151	1.010	0.008	1.99%
GSE31863	0.140	-0.268	0.548	0.501	2.02%
GSE24185	1.129	0.702	1.556	< 0.001	1.99%
GSE22219	0.913	0.625	1.201	< 0.001	2.20%
GSE19783	0.657	0.268	1.046	0.001	2.05%
GSE20194	0.637	0.392	0.881	< 0.001	2.25%
GSE10810	0.732	-0.013	1.477	0.054	1.48%
GSE8757	0.116	-0.202	0.435	0.474	2.16%
GSE7390	0.721	0.415	1.028	< 0.001	2.17%
GSE129559	0.754	0.323	1.186	0.001	1.99%
GSE130786	-0.611	-0.999	-0.224	0.002	2.06%
GSE103744	0.941	0.597	1.285	< 0.001	2.12%
GSE86166	0.699	0.453	0.945	< 0.001	2.25%
GSE93601	0.839	0.637	1.040	< 0.001	2.30%
GSE59246	0.865	0.383	1.348	< 0.001	1.90%
GSE71258	0.987	0.521	1.453	< 0.001	1.93%
GSE61304	1.290	0.698	1.883	< 0.001	1.72%
GSE29044	1.020	0.460	1.581	< 0.001	1.78%
GSE37181	0.835	0.370	1.300	< 0.001	1.93%
GSE48390	0.442	-0.021	0.905	0.061	1.94%
GSE43365	1.003	0.481	1.525	< 0.001	1.84%
GSE45255	0.806	0.442	1.170	< 0.001	2.09%
GSE35629	-0.019	-0.585	0.547	0.947	1.77%
GSE29210	-0.123	-0.414	0.168	0.407	2.20%
GSE34138	1.050	0.721	1.379	< 0.001	2.14%
GSE10893	-0.227	-0.514	0.061	0.123	2.20%
GSE16987	1.214	0.815	1.614	< 0.001	2.04%
GSE22597	0.957	0.498	1.416	< 0.001	1.94%
GSE26639	0.658	0.384	0.932	< 0.001	2.22%
GSE26304	1.332	0.832	1.832	< 0.001	1.88%
GSE20271	0.755	0.449	1.060	< 0.001	2.18%
GSE20624	-0.157	-0.513	0.200	0.389	2.10%
GSE21653	0.947	0.690	1.204	< 0.001	2.24%
GSE10510	0.163	-0.167	0.493	0.333	2.14%
GSE1561	0.502	-0.070	1.074	0.085	1.76%
GSE2034	0.081	-0.180	0.342	0.544	2.23%
GSE17907	0.158	-0.416	0.732	0.589	1.75%
GSE19615	0.854	0.464	1.245	< 0.001	2.05%
**Total (95% CI)**	**0.634**	**0.488**	**0.780**	**< 0.001**	**100%**

Std diff in means and 95% CI

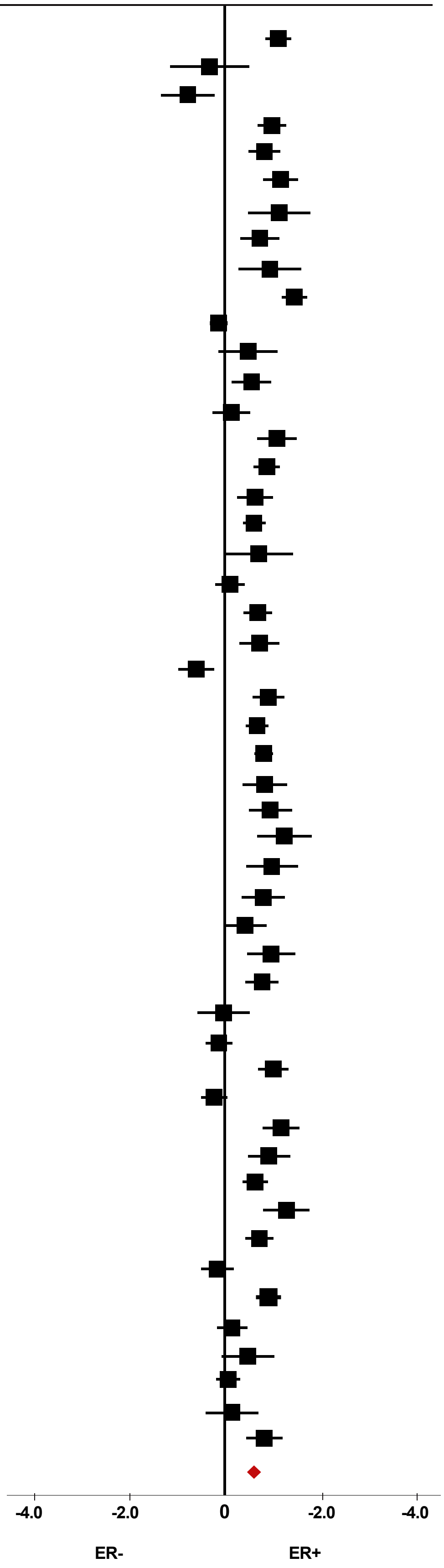

Heterogeneity: Q-value = 399.48; df = 49 (p < 0.001); I^2 = 87.73%

Supplementary Figure S2. ***Cont.***

2B)

Study	Std diff in means	Lower limit	Upper limit	p-Value	Weight
GSE89116	0.202	-0.668	1.073	0.649	1.64%
GSE76360	-0.395	-1.018	0.227	0.213	2.33%
GSE49481	0.568	0.313	0.823	< 0.001	3.70%
GSE59590	0.648	0.329	0.966	< 0.001	3.47%
GSE65194	1.205	0.830	1.580	< 0.001	3.25%
GSE60785	1.229	0.613	1.845	< 0.001	2.36%
GSE37126	0.478	-0.104	1.061	0.107	2.47%
GSE41998	1.197	0.932	1.462	< 0.001	3.67%
GSE25307	0.050	-0.122	0.222	0.568	3.95%
GSE31863	-0.049	-0.403	0.305	0.785	3.33%
GSE24185	1.059	0.636	1.482	< 0.001	3.07%
GSE20194	0.453	0.213	0.693	< 0.001	3.75%
GSE129559	0.754	0.323	1.186	0.001	3.03%
GSE130786	-0.122	-0.502	0.258	0.530	3.23%
GSE86166	0.597	0.366	0.828	< 0.001	3.78%
GSE59246	0.711	0.254	1.168	0.002	2.93%
GSE71258	1.131	0.654	1.608	< 0.001	2.86%
GSE61304	0.868	0.256	1.479	0.005	2.37%
GSE29044	0.831	0.323	1.340	0.001	2.74%
GSE43365	0.533	0.124	0.943	0.011	3.12%
GSE45255	0.391	0.045	0.736	0.027	3.37%
GSE34138	0.486	0.167	0.806	0.003	3.47%
GSE16987	0.604	0.275	0.932	< 0.001	3.43%
GSE26639	0.884	0.606	1.162	< 0.001	3.62%
GSE26304	0.817	0.379	1.256	< 0.001	3.01%
GSE20271	0.674	0.371	0.976	< 0.001	3.53%
GSE20624	-0.185	-0.553	0.183	0.326	3.28%
GSE21653	0.907	0.653	1.161	< 0.001	3.70%
GSE10510	0.141	-0.183	0.465	0.395	3.45%
GSE1561	0.647	0.044	1.249	0.035	2.40%
GSE17907	0.498	-0.093	1.089	0.099	2.44%
GSE19615	0.576	0.201	0.951	0.003	3.25%
**Total (95% CI)**	**0.576**	**0.433**	**0.718**	**<0.001**	**100%**

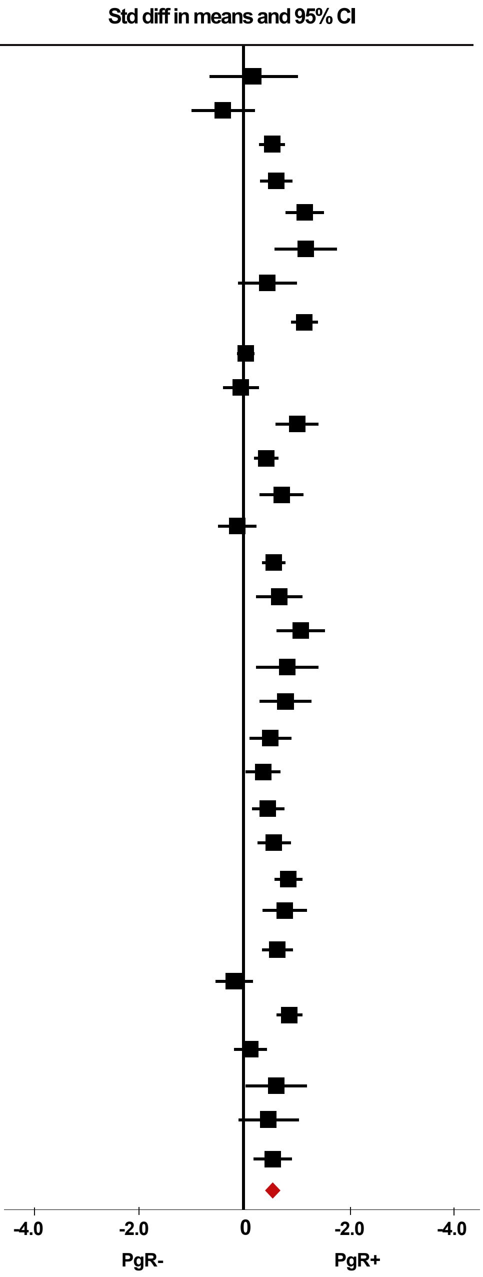

Heterogeneity: Q-value = 158.02; df = 31 (p < 0.001); I^2 = 80.38%

Supplementary Figure S2. ***Cont.***

2C)

Study	Std diff in means	Lower limit	Upper limit	p-Value	Weight
GSE76360	0.122	-0.718	0.962	0.776	1.75%
GSE49481	0.250	-0.072	0.572	0.128	6.04%
GSE59590	0.371	-0.100	0.841	0.122	4.08%
GSE60785	0.689	-0.112	1.491	0.092	1.89%
GSE37126	-0.396	-1.224	0.432	0.348	1.79%
GSE41998	0.344	-0.119	0.808	0.145	4.16%
GSE25307	0.434	0.170	0.699	0.001	7.01%
GSE31863	-0.134	-0.602	0.335	0.576	4.10%
GSE24185	0.460	-0.258	1.178	0.209	2.25%
GSE20194	0.278	-0.051	0.607	0.098	5.93%
GSE130786	0.460	-0.094	1.015	0.104	3.30%
GSE86166	0.220	-0.175	0.614	0.275	4.99%
GSE71258	0.800	0.097	1.502	0.026	2.33%
GSE29044	0.358	-0.323	1.039	0.303	2.45%
GSE43365	0.064	-0.475	0.602	0.817	3.43%
GSE45255	-0.284	-0.786	0.218	0.268	3.76%
GSE34138	-0.057	-0.426	0.312	0.761	5.34%
GSE10893	-0.224	-0.666	0.219	0.322	4.40%
GSE16987	0.186	-0.202	0.575	0.347	5.06%
GSE26639	0.698	0.328	1.067	<0.001	5.33%
GSE26304	0.253	-0.397	0.902	0.446	2.63%
GSE20271	0.009	-0.482	0.500	0.972	3.87%
GSE20624	-0.203	-0.750	0.345	0.468	3.36%
GSE21653	0.487	0.013	0.961	0.044	4.05%
GSE10510	-0.163	-0.771	0.445	0.600	2.90%
GSE1561	0.251	-0.552	1.054	0.540	1.88%
GSE19615	-0.842	-1.635	-0.048	0.038	1.92%
**Total (95% CI)**	**0.187**	**0.065**	**0.309**	**0.003**	**100%**

Heterogeneity: Q-value = 42.21; df = 26 ($p < 0.02$); $I^2 = 38.41\%$

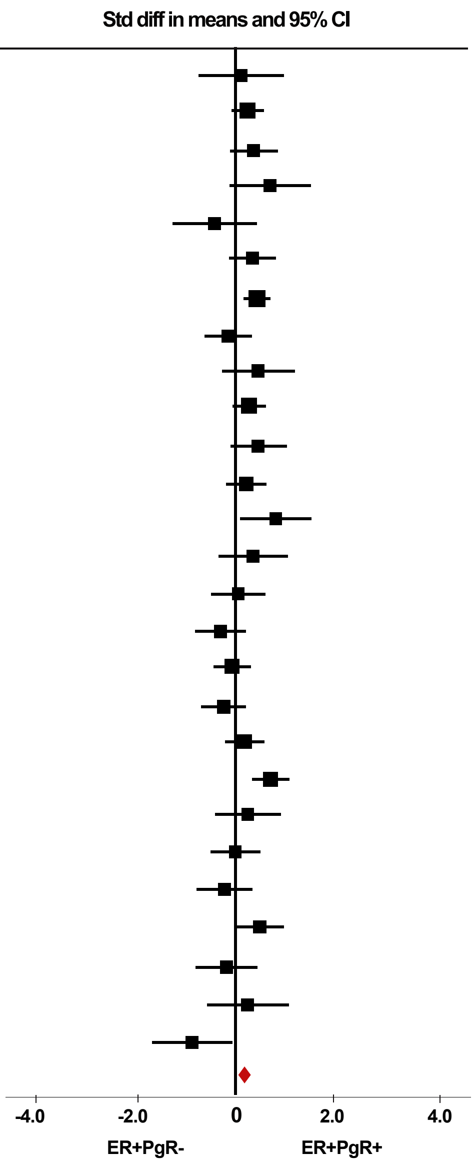

Supplementary Figure S2. ***Cont.***

2D)

Study	Std diff in means	Lower limit	Upper limit	p-Value	Weight
GSE80999	0.287	-0.040	0.613	0.085	4.56%
GSE89116	0.844	-0.060	1.748	0.067	1.00%
GSE49481	0.210	-0.147	0.568	0.249	4.13%
GSE59590	0.119	-0.312	0.550	0.587	3.27%
GSE65194	0.630	0.277	0.983	< 0.001	4.18%
GSE60785	0.288	-0.736	1.312	0.582	0.80%
GSE37126	0.225	-0.450	0.900	0.513	1.66%
GSE41998	0.717	0.322	1.112	< 0.001	3.66%
GSE31364	0.408	-0.194	1.009	0.184	2.00%
GSE20711	0.218	-0.241	0.677	0.352	3.00%
GSE24185	0.852	0.210	1.494	0.009	1.80%
GSE20194	0.064	-0.224	0.351	0.663	5.19%
GSE126870	-0.206	-0.644	0.233	0.358	3.19%
GSE129559	0.410	-0.058	0.877	0.086	2.93%
GSE86166	0.105	-0.188	0.398	0.483	5.10%
GSE59246	0.008	-0.520	0.536	0.976	2.45%
GSE71258	0.122	-0.384	0.629	0.636	2.61%
GSE29044	-0.297	-0.782	0.189	0.231	2.77%
GSE37181	0.070	-0.430	0.570	0.784	2.65%
GSE43365	0.030	-0.549	0.610	0.918	2.13%
GSE45255	-0.019	-0.362	0.324	0.916	4.32%
GSE35629	-0.284	-0.901	0.333	0.367	1.92%
GSE10893	0.095	-0.230	0.420	0.566	4.59%
GSE16987	-0.492	-0.919	-0.065	0.024	3.31%
GSE22597	0.029	-0.423	0.482	0.899	3.06%
GSE26639	0.367	0.093	0.641	0.009	5.42%
GSE26304	0.072	-0.364	0.508	0.746	3.21%
GSE20271	0.347	-0.070	0.765	0.103	3.41%
GSE20624	0.103	-0.305	0.510	0.622	3.51%
GSE21653	0.249	-0.139	0.637	0.209	3.74%
GSE17907	0.047	-0.991	1.085	0.929	0.78%
GSE19615	0.261	-0.135	0.656	0.196	3.65%
**Total (95% CI)**	**0.174**	**0.002**	**0.079**	**< 0.001**	**100%**

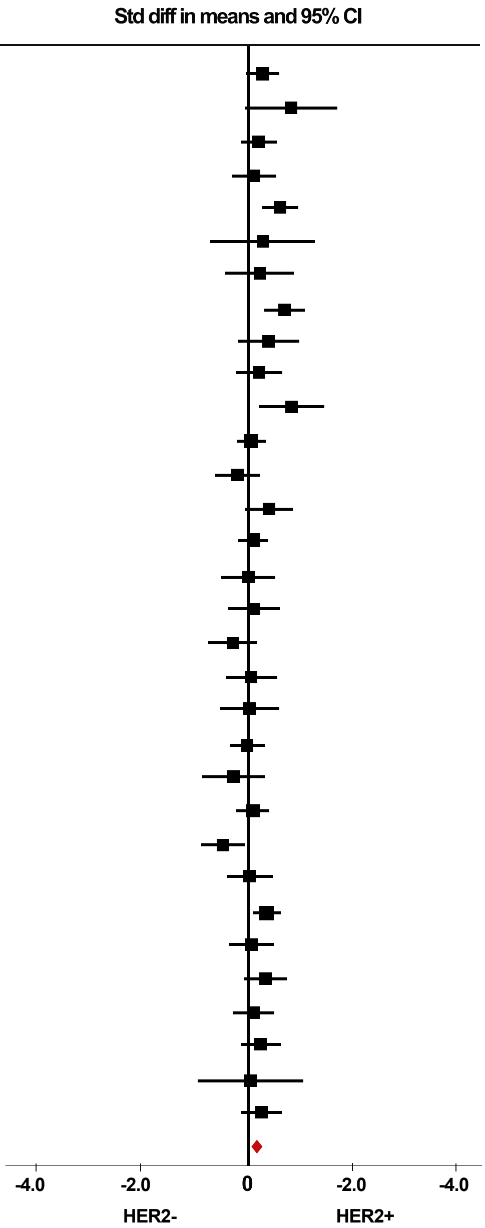

Heterogeneity: Q-value = 46.93; df = 31 ($p < 0.03$); I^2 = 33.95%

Supplementary Figure S2. *AR* mRNA expression is higher in BC patients expressing either ER, PgR, ER+/PgR+, and HER2. Results from the primary analysis. Forest plots of SMD showing *AR* mRNA levels associated with ER+ **(A)**, PgR+ **(B)**, ER+/PgR+ **(C)**, and HER2+ status **(D)**. SMD for each dataset are represented by the squares, and the horizontal line crossing the square represents the 95% CI. The red diamonds represent the estimated overall effect.

Study	Std diff in means	Lower limit	Upper limit	p-Value	Weight
GSE76360	0.122	-0.718	0.962	0.776	1.62%
GSE49481	0.250	-0.072	0.572	0.128	6.58%
GSE59590	0.371	-0.100	0.841	0.122	4.11%
GSE60785	0.689	-0.112	1.491	0.092	1.75%
GSE37126	-0.396	-1.224	0.432	0.348	1.66%
GSE41998	0.344	-0.119	0.808	0.145	4.20%
GSE25307	0.434	0.170	0.699	0.001	7.96%
GSE31863	-0.134	-0.602	0.335	0.576	4.14%
GSE24185	0.460	-0.258	1.178	0.209	2.12%
GSE20194	0.278	-0.051	0.607	0.098	6.43%
GSE130786	0.460	-0.094	1.015	0.104	3.23%
GSE86166	0.220	-0.175	0.614	0.275	5.21%
GSE71258	0.800	0.097	1.502	0.026	2.20%
GSE29044	0.358	-0.323	1.039	0.303	2.32%
GSE43365	0.064	-0.475	0.602	0.817	3.37%
GSE45255	-0.284	-0.786	0.218	0.268	3.75%
GSE34138	-0.057	-0.426	0.312	0.761	5.65%
GSE10893	-0.224	-0.666	0.219	0.322	4.48%
GSE16987	0.186	-0.202	0.575	0.347	5.30%
GSE26639	0.698	0.328	1.067	< 0.001	5.64%
GSE26304	0.253	-0.397	0.902	0.446	2.51%
GSE20271	0.009	-0.482	0.500	0.972	3.87%
GSE20624	-0.203	-0.750	0.345	0.468	3.29%
GSE21653	0.487	0.013	0.961	0.044	4.07%
GSE10510	-0.163	-0.771	0.445	0.600	2.79%
GSE1561	0.251	-0.552	1.054	0.540	1.75%
**Total (95% CI)**	**0.210**	0.096	**0.323**	**< 0.001**	**100%**

Heterogeneity: Q-value = 35.38; df = 25 (p = 0.081); I^2 = 29.34%

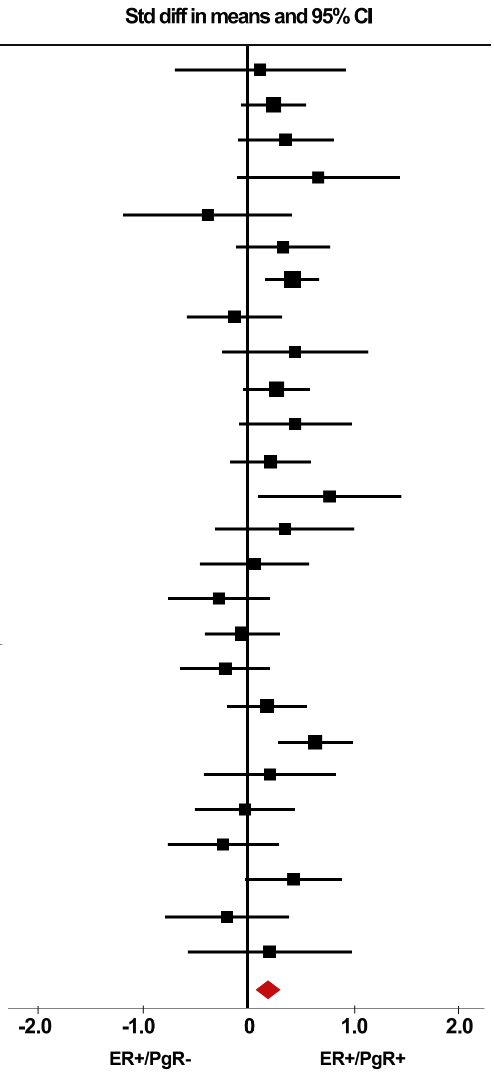

Supplementary Figure S3. Forest plot showing that *AR* mRNA expression is higher in BC patients expressing ER+/PgR+ receptors. Results after sensitivity analysis. SMD for each dataset are represented by the squares, and the horizontal line crossing the square represents the 95% CI. The red diamond represents the estimated overall effect.

4A)

Study	Std diff in means	Lower limit	Upper limit	p-Value	Weight
GSE80999	0.621	0.252	0.990	0.001	6.95%
GSE49481	1.903	1.469	2.336	<0.001	6.91%
GSE59590	4.908	4.074	5.742	<0.001	6.51%
GSE60785	3.986	2.656	5.316	<0.001	5.81%
GSE25307	0.231	-0.013	0.476	0.063	7.01%
GSE59246	2.808	1.780	3.835	<0.001	6.26%
GSE20624	-0.755	-1.203	-0.306	0.001	6.89%
GSE35629	0.064	-0.707	0.835	0.871	6.59%
GSE10893	-0.881	-1.236	-0.525	<0.001	6.96%
GSE78958	2.254	1.961	2.547	<0.001	6.99%
GSE48390	1.594	0.751	2.437	<0.001	6.50%
GSE34138	2.015	1.559	2.472	<0.001	6.89%
GSE21653	3.043	2.593	3.494	<0.001	6.89%
GSE126870	2.818	1.853	3.783	<0.001	6.34%
GSE104549	1.945	1.112	2.779	<0.001	6.51%
**Total (95% CI)**	**1.724**	**0.963**	**2.484**	**<0.001**	**100%**

Heterogeneity: Q-value = 532.92; df = 14 ($p < 0.001$); $I^2 = 97.37\%$

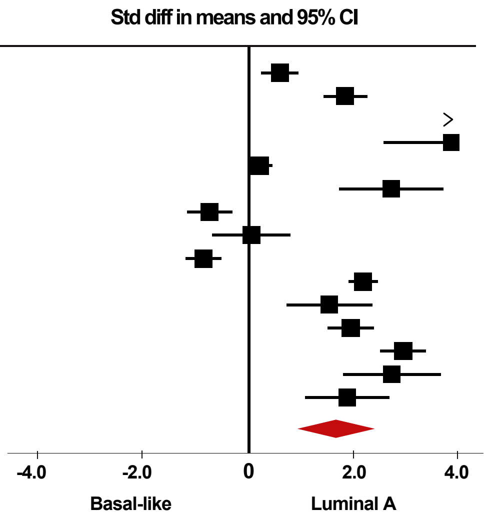

4B)

Study	Std diff in means	Lower limit	Upper limit	p-Value	Weight
GSE80999	0.678	0.275	1.081	0.001	7.22%
GSE49481	1.746	1.322	2.171	<0.001	7.18%
GSE59590	3.356	2.583	4.129	<0.001	6.49%
GSE60785	4.045	2.618	5.472	<0.001	4.88%
GSE25307	-0.085	-0.354	0.184	0.536	7.39%
GSE126870	1.929	0.917	2.940	<0.001	5.91%
GSE104549	2.775	1.867	3.684	<0.001	6.17%
GSE59246	1.661	0.747	2.575	<0.001	6.16%
GSE35629	0.222	-0.581	1.024	0.588	6.42%
GSE10893	-0.269	-0.658	0.120	0.175	7.24%
GSE20624	0.084	-0.413	0.581	0.741	7.06%
GSE78958	1.676	1.268	2.083	<0.001	7.21%
GSE48390	1.059	0.207	1.911	0.015	6.31%
GSE34138	1.179	0.731	1.626	<0.001	7.15%
GSE21653	1.493	1.088	1.898	<0.001	7.21%
**Total (95% CI)**	**1.351**	**0.820**	**1.883**	**<0.001**	**100%**

Heterogeneity: Q-value = 219.49; df = 14 ($p < 0.001$); $I^2 = 93.62\%$

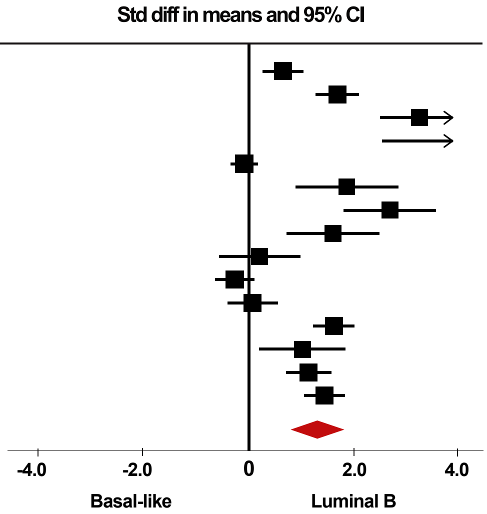

4C)

Study	Std diff in means	Lower limit	Upper limit	p-Value	Weight
GSE80999	0.825	0.336	1.315	0.001	7.25%
GSE49481	2.366	1.711	3.020	<0.001	6.97%
GSE59590	2.734	1.991	3.477	<0.001	6.80%
GSE60785	3.529	2.116	4.941	<0.001	5.29%
GSE25307	0.431	0.148	0.713	0.003	7.50%
GSE126870	1.495	0.368	2.621	0.009	5.95%
GSE104549	2.047	0.831	3.263	0.001	5.74%
GSE59246	1.433	0.328	2.538	0.011	6.00%
GSE35629	-0.134	-1.029	0.762	0.770	6.48%
GSE10893	-0.628	-1.074	-0.183	0.006	7.31%
GSE20624	-0.391	-0.955	0.172	0.173	7.13%
GSE78958	1.848	1.449	2.248	<0.001	7.37%
GSE48390	1.469	0.557	2.380	0.002	6.44%
GSE34138	1.044	0.224	1.863	0.013	6.64%
GSE21653	2.319	1.757	2.881	<0.001	7.13%
**Total (95% CI)**	**1.305**	**0.719**	**1.891**	**<0.001**	**100%**

Heterogeneity: Q-value = 190.02; df = 14 ($p < 0.001$); $I^2 = 92.63\%$

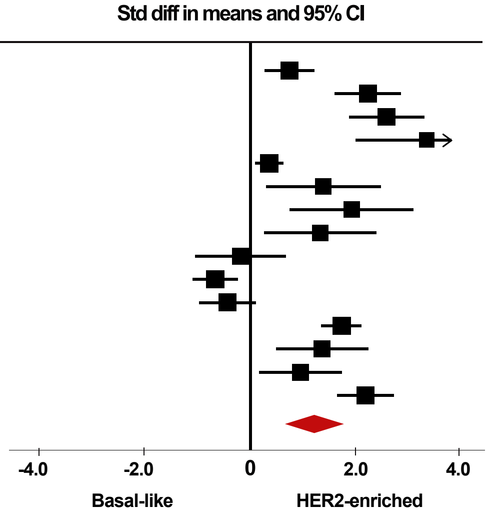

Supplementary Figure S4. *AR* mRNA level is significantly increased in patients categorized within the less aggressive intrinsic molecular subtypes. Results from the primary analysis. Forest plots of SMD comparing *AR* mRNA levels in BC patients classified as Basal-like subtype Vs Luminal A **(A)**, Luminal B **(B)**, and HER2-enriched **(C)**. SMD for each dataset are represented by the squares, and the horizontal line crossing the square represents the 95% CI. The red diamonds represent the estimated overall effect.

5A)

Study	Std diff in means	Lower limit	Upper limit	p-Value	Weight
GSE65194	2.043	1.459	2.627	<0.001	7.55%
GSE20711	0.978	0.448	1.509	<0.001	7.68%
GSE45827	4.261	3.549	4.974	<0.001	7.20%
GSE34138	1.174	0.840	1.508	<0.001	8.08%
GSE21653	1.016	0.736	1.297	<0.001	8.16%
GSE80999	1.434	1.136	1.731	<0.001	8.13%
GSE49481	1.310	0.952	1.668	<0.001	8.03%
GSE59590	1.177	0.756	1.599	<0.001	7.92%
GSE60785	1.908	1.139	2.677	<0.001	7.04%
GSE59246	2.038	1.255	2.821	<0.001	7.00%
GSE35629	-0.047	-0.770	0.675	0.898	7.17%
GSE10893	-0.314	-0.609	-0.019	0.037	8.14%
GSE20624	-0.113	-0.534	0.307	0.598	7.92%
**Total (95% CI)**	**1.268**	**0.754**	**1.782**	**<0.001**	**100%**

Heterogeneity: Q-value = 228.77; df = 12 ($p < 0.001$); I^2 = 94.75%

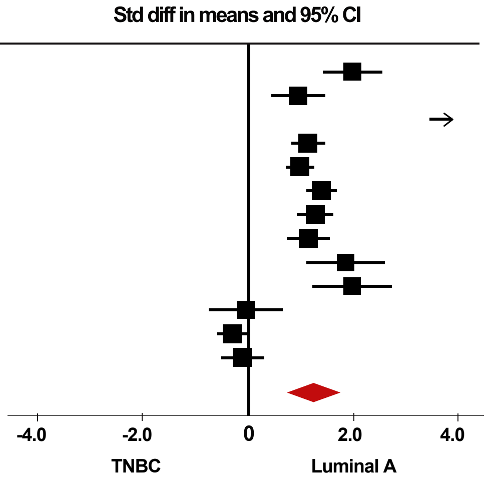

5B)

Study	Std diff in means	Lower limit	Upper limit	p-Value	Weight
GSE65194	3.648	2.885	4.411	<0.001	10.01%
GSE37126	1.510	0.489	2.530	0.004	9.36%
GSE21653	0.906	0.373	1.438	0.001	10.47%
GSE80999	2.287	1.696	2.879	<0.001	10.37%
GSE49481	2.359	1.701	3.017	<0.001	10.23%
GSE59590	1.080	0.407	1.753	0.002	10.20%
GSE59246	2.036	0.788	3.284	0.001	8.73%
GSE35629	-0.857	-1.760	0.045	0.063	9.67%
GSE10893	-0.105	-0.562	0.351	0.651	10.60%
GSE20624	0.078	-0.523	0.678	0.800	10.35%
**Total (95% CI)**	**1.282**	**0.464**	**2.100**	**0.002**	**100%**

Heterogeneity: Q-value = 131.66; df = 9 ($p < 0.001$); I^2 = 93.16%

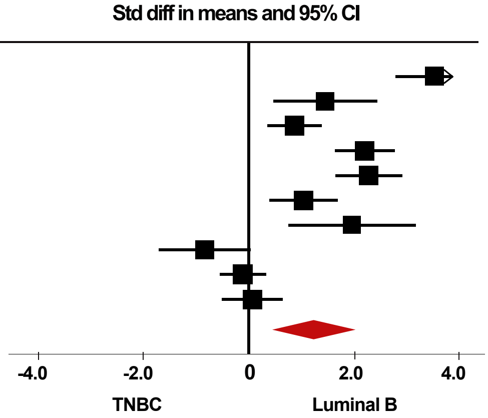

5C)

Study	Std diff in means	Lower limit	Upper limit	p-Value	Weight
GSE65194	2.195	1.602	2.788	<0.001	9.41%
GSE20711	0.836	0.274	1.398	0.004	9.53%
GSE45827	2.216	1.620	2.811	<0.001	9.40%
GSE21653	1.127	0.610	1.644	<0.001	9.69%
GSE80999	1.361	0.794	1.927	<0.001	9.51%
GSE49481	1.634	0.941	2.327	<0.001	9.01%
GSE59590	0.794	0.057	1.530	0.035	8.83%
GSE59246	1.505	0.409	2.602	0.007	7.28%
GSE35629	0.219	-0.678	1.116	0.632	8.14%
GSE10893	-0.153	-0.625	0.318	0.524	9.84%
GSE20624	-0.076	-0.676	0.525	0.805	9.38%
**Total (95% CI)**	**1.055**	**0.532**	**1.578**	**<0.001**	**100%**

Heterogeneity: Q-value = 76.25; df = 10 ($p < 0.001$); I^2 = 88.88%

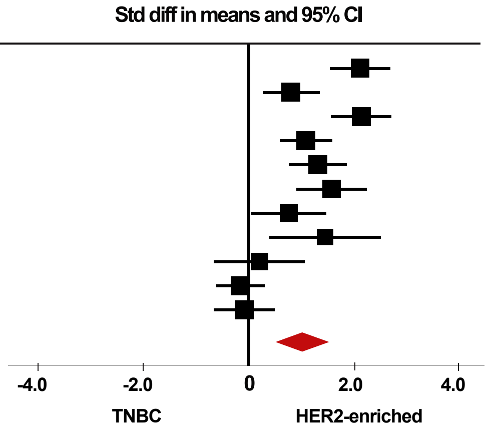

Supplementary Figure S5. *AR* mRNA level is significantly increased in patients categorized with less aggressive IHC-surrogate subtypes. Results from the primary analysis. Forest plots of SMD comparing *AR* mRNA levels in BC patients classified as TNBC subtype Vs Luminal A **(A)**, Luminal B **(B)** and HER2-enriched subtypes **(C)**. SMD for each dataset are represented by the squares, and the horizontal line crossing the square repre-sents the 95% CI. The red diamonds represent the estimated overall effect.

6A)

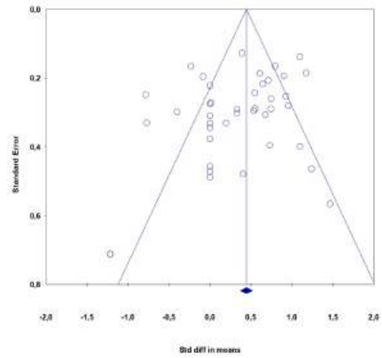

Egger's test: -1.52; 95%CI: -3.25 to 0.20; *p* = 0.082

6B)

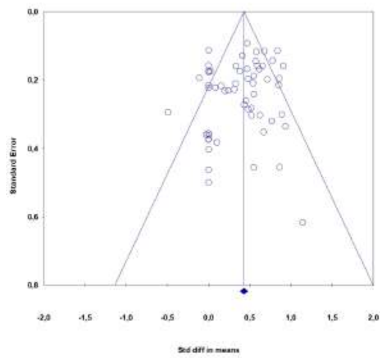

Egger's test: -0.72; 95%CI: -1.79 to 0.33; *p* = 0.177

6C)

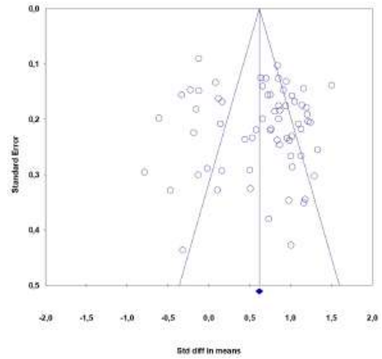

Egger's test: 0.71; 95%CI: -1.41 to 2.84; *p* = 0.504

6D)

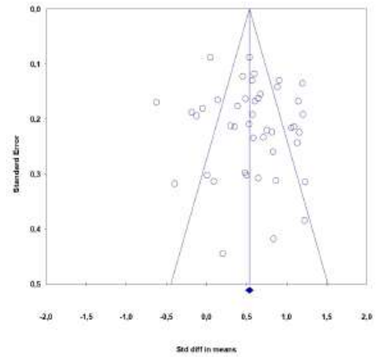

Egger's test: 0.81; 95%CI: -1.18 to 2.81; *p* = 0.414

6E)

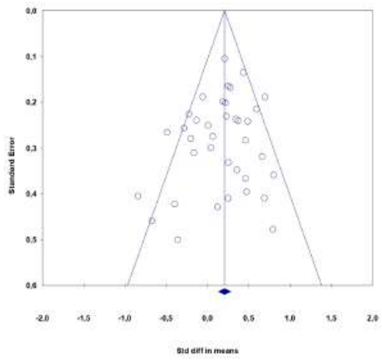

Egger's test: -0.68; 95%CI: -1.85 to 0.47; *p* = 0.239

6F)

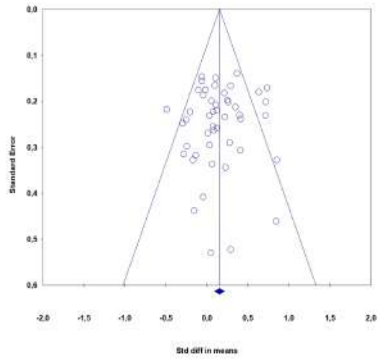

Egger's test: -0.31; 95%CI: -1.64 to 1.02; *p* = 0.641

Supplementary Figure S6. Funnel plots for main meta-analyses in the primary analysis. **(A)** Association between *AR* mRNA level in BC patients with histological grade 3 Vs BC patients with histological grade 1, and **(B)** histological grade 2 **(C).** *AR* mRNA levels associated with ER **(D)**, PgR **(E)**, ER/PgR **(F)**, and HER2 status **(G).**

7A)

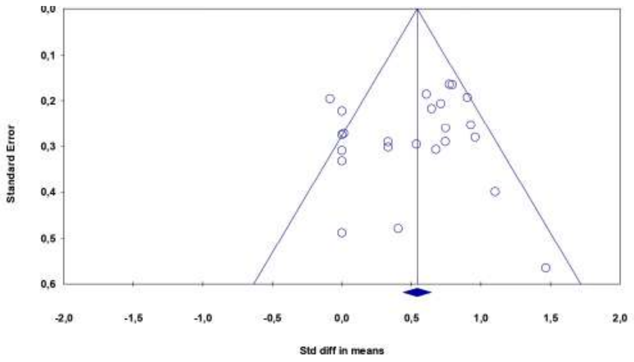

Egger's test: -0.68; 95%CI: -2.89 to 1.52; *p* = 0.526

7B)

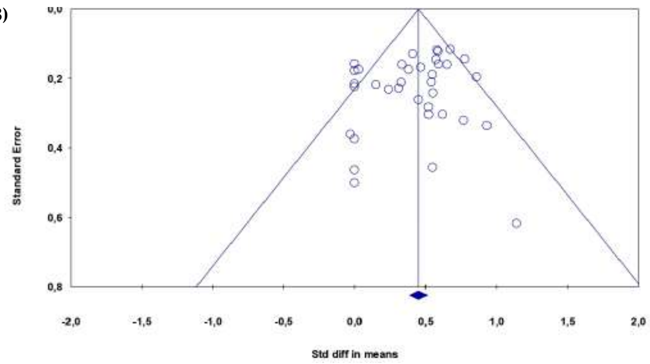

Egger's test: -0.77; 95%CI: -2.02 to 0.47; *p* = 0.217

7C)

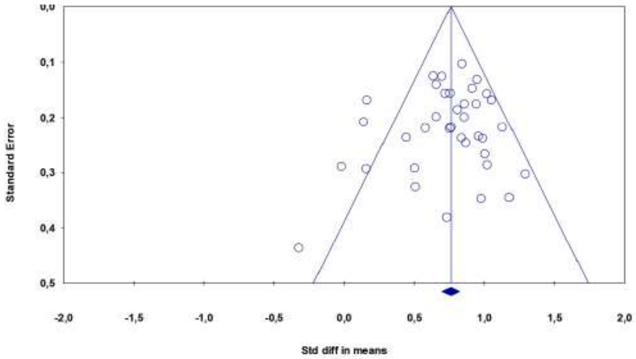

Egger's test: -0.59; 95%CI: -2.01 to 0.82; *p* = 0.398

7D)

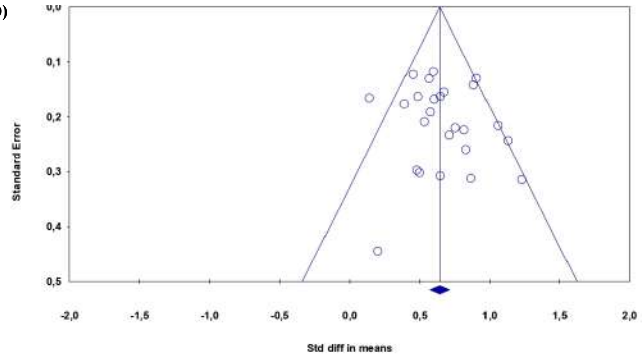

Egger's test: 0.68; 95%CI: -0.97 to 2.34; *p* = 0.403

7E)

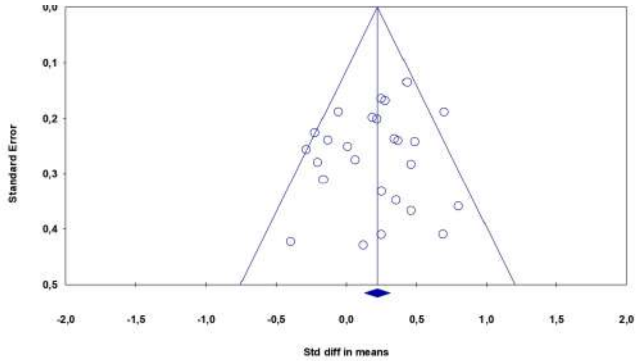

Egger's test: -0.61; 95%CI: -2.21 to 0.97; *p* = 0.432

7F)

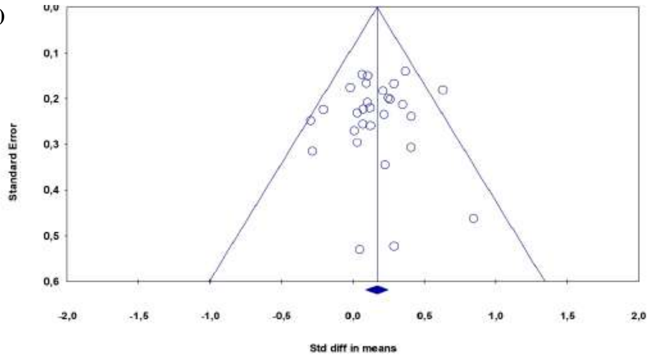

Egger's test: -0.26; 95%CI: -1.54 to 1.01; *p* = 0.676

Supplementary Figure S7. Funnel plots for main meta-analyses after sensitivity analysis. **(A)** Association between *AR* mRNA level in BC patients with histological grade 3 Vs BC patients with histological grade 1, and **(B)** histological grade 2 **(C).** *AR* mRNA levels associated with ER **(D)**, PgR **(E)**, ER/PgR **(F)**, and HER2 status **(G).**

8A)

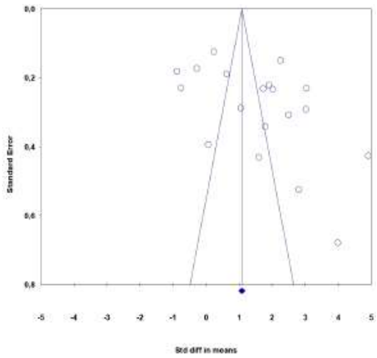

Egger's test: -7.00; 95%CI: -0.08 to 14.09; *p* = 0.052

8B)

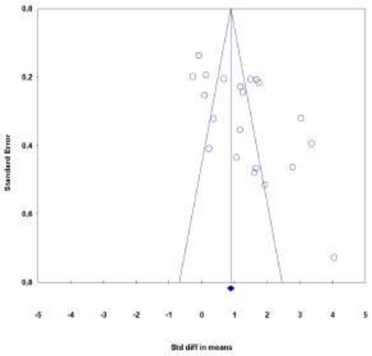

Egger's test: 5.69; 95%CI: 1.84 to 9.55; *p* = 0.005

8C)

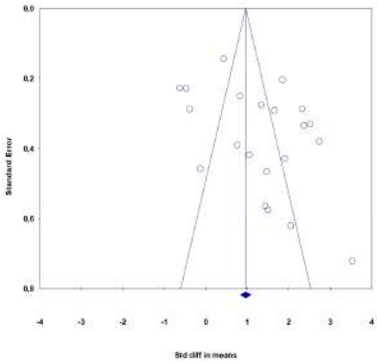

Egger's test: 3.85; 95%CI: -0.18 to 7.90; *p* = 0.060

8D)

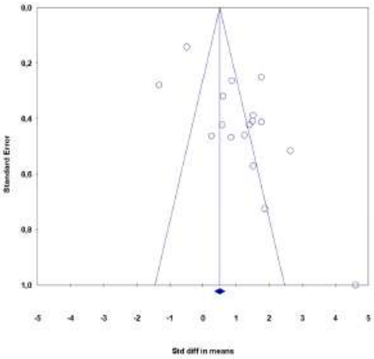

Egger's test: 5.22; 95%CI: 2.16 to 8.28; *p* = 0.002

8E)

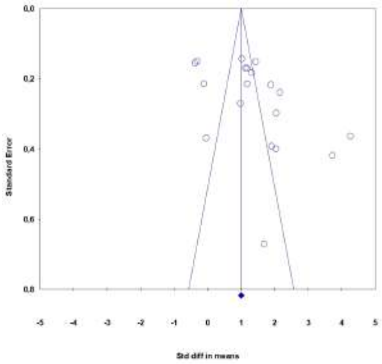

Egger's test: 6.33; 95%CI: 0.65 to 12.0; *p* = 0.030

8F)

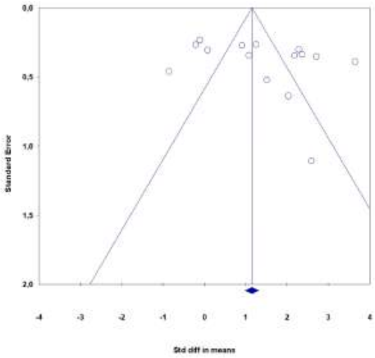

Egger's test: 4.21; 95%CI: -2.52 to 10.95; *p* = 0.200

8G)

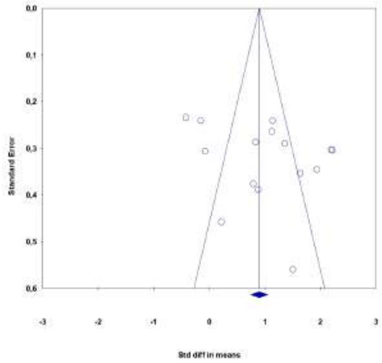

Egger's test: 4.24; 95%CI: -3.22 to 11.71; *p* = 0.241

Supplementary Figure S8. Funnel plots for main meta-analyses in the primary analysis. Association between *AR* mRNA level in BC patients classified with the intrinsic molecular subtypes Basal-like Vs Luminal A **(A)**, Luminal B **(B)**, HER2-enriched **(C)**, and Normal-like **(D)**. Association between *AR* mRNA level in BC patients classified with the IHC-surrogate subtypes TNBC Vs Luminal A **(E)**, Luminal B **(F)**, and HER2-enriched **(G)**.

9A)

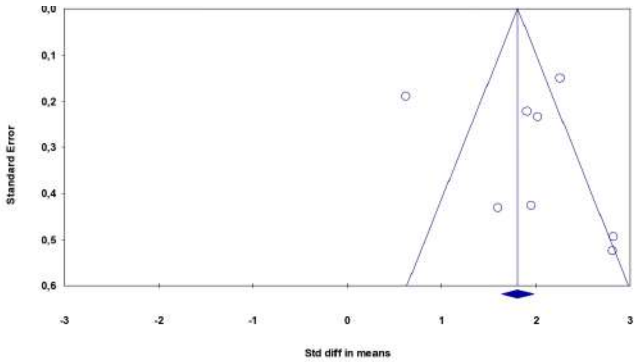

Egger's test: 1.36; 95%CI: -4.99 to 7.71; *p* = 0.619

9B)

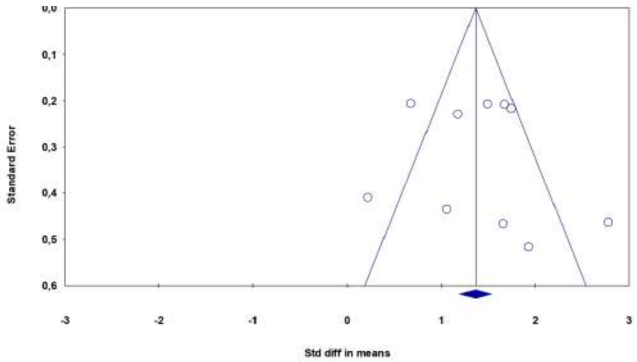

Egger's test: 0.81; 95%CI: -3.67 to 5.30; *p* = 0.686

9C)

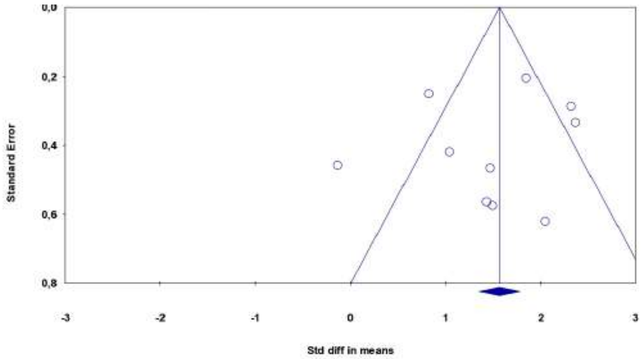

Egger's test: -0.10; 95%CI: -5.54 to 3.33; *p* = 0.580

9D)

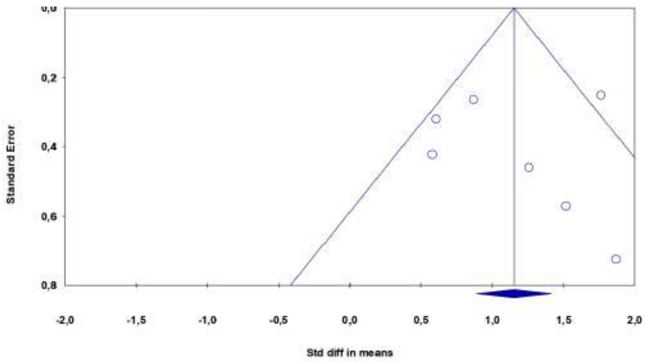

Egger's test: 0.18; 95%CI: -4.60 to 4.96; *p* = 0.926

9E)

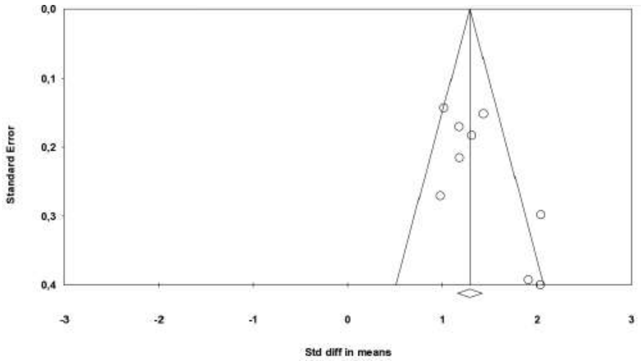

Egger's test: 2.88; 95%CI: -0.14 to 5.91; *p* = 0.058

9F)

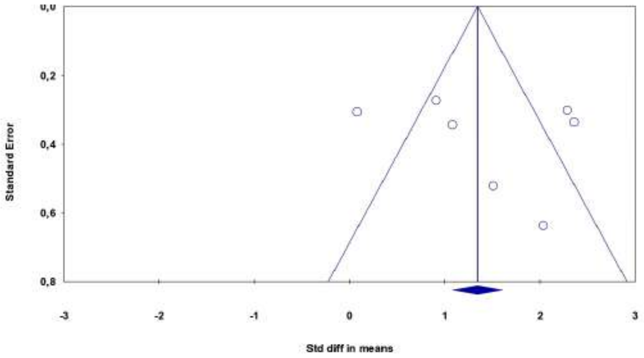

Egger's test: 2.42; 95%CI: -8.41 to 13.25; *p* = 0.590

9G)

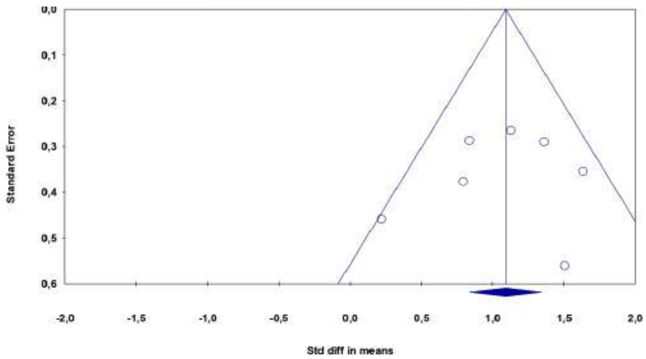

Egger's test: -0.68; 95%CI: -6.23 to 4.87; *p* = 0.765

Supplementary Figure S9. Funnel plots for main meta-analyses after sensitivity analysis. Association between *AR* mRNA level in BC patients classified with the intrinsic molecular subtypes Basal-like Vs Luminal A **(A)**, Luminal B **(B)**, HER2-enriched **(C)**, and Normal-like **(D)**. Association between *AR* mRNA level in BC patients classified with the IHC-surrogate subtypes TNBC Vs Luminal A **(E)**, Luminal B **(F)**, and HER2-enriched **(G)**.

Table S1. Overview of the datasets selected from GEO for clinico-pathological analyses.

GEO Dataset	Year	Country	Platform	AR gene ID	Microarray ID	Reference
GSE80999	2017	Norway	Agilent-028004 SurePrint G3 Human GE 8x60K Microarray	A_23_P113111	GPL14550	[1–3]
GSE89116	2016	India	Illumina HumanHT-12 V3.0 expression beadchip	ILMN_1767351	GPL6947	[4]
GSE76360	2016	USA	Illumina HumanHT-12 V3.0 expression beadchip	ILMN_1767351	GPL6947	[5]
GSE49481	2015	Denmark	Agilent-029949 Custom SurePrint G3 Human GE 8x60K	A_23_P113111	GPL15931	[6]
GSE59590	2015	Italy	Illumina HumanHT-12_V3_0_R1_Bioconductor_2.11	ILMN_1767351	GPL17518	[7]
GSE65194	2015	France	Affymetrix Human Genome U133 Plus 2.0 Array	211110_s_at	GPL570	[8–10]
GSE60785	2014	Sweden	Illumina HumanHT-12 V4.0 expression beadchip	ILMN_1767351	GPL10558	[11]
GSE42568	2013	Ireland	Affymetrix Human Genome U133 Plus 2.0 Array	211110_s_at	GPL570	[12]
GSE37126	2013	Brazil	Agilent-014850 Whole Human Genome Microarray 4x44K G4112F	A_23_P113111	GPL6480	[13,14]
GSE41998	2013	USA	Affymetrix Human Genome U133A 2.0 Array	211110_s_at	GPL571	[15]
GSE25307	2012	Sweden	SWEGENE H_v2.1.1 55K	26950	GPL5345	[16]
GSE31364	2012	Netherlands	Agendia_human_DiscoverPrint_v1	Agendia_DiscoverPrint_v1_probe_14025	GPL14378	[17]
GSE20711	2011	Canada	Affymetrix Human Genome U133 Plus 2.0 Array	211110_s_at	GPL570	[18]
GSE31863	2011	Sweden	SWEGENE Human_v2.1.1 55K_condensed	H200011018	GPL14374	[19]
GSE24185	2011	USA	Affymetrix Human Genome U133A Array	211621_at	GPL96	[20]
GSE22219	2011	United Kingdom	Illumina humanRef-8 v1.0 expression beadchip	5290131	GPL6098	[21]
GSE20194	2010	China	Affymetrix Human Genome U133A Array	211621_at	GPL96	[22,23]

GSE10810	2009	Spain	Affymetrix Human Genome U133 Plus 2.0 Array	211110_s_at	GPL570	[24]
GSE8757	2007	United Kingdom	Human 30K 60-mer oligo array	27143	GPL5737	[25,26]
GSE7390	2007	Canada	Affymetrix Human Genome U133A Array	211621_at	GPL96	[27,28]
GSE129559	2019	USA	Affymetrix Human Genome U133A Array	211621_at	GPL96	[29]
GSE130786	2019	USA	Agilent-014850 Whole Human Genome Microarray 4x44K G4112F	A_23_P113111	GPL6480	[30]
GSE103744	2018	Sweden	Illumina HumanHT-12 V4.0 expression beadchip	ILMN_1767351	GPL10558	[31]
GSE86166	2017	USA	Rosetta/Merck Human RSTA Custom Affymetrix 2.0 microarray	merck-NM_000044_a_at	GPL15048	[32]
GSE93601	2017	USA	Custom Affymetrix Human Transcriptome Array	TC0X00308	GPL22920	[33]
GSE59246	2016	USA	Agilent-028004 SurePrint G3 Human GE 8x60K Microarray	990	GPL13607	[34]
GSE71258	2015	USA	Affymetrix Human Genome U133 Plus 2.0 Array	211110_s_at	GPL570	[35]
GSE61304	2015	Singapore	Affymetrix Human Genome U133 Plus 2.0 Array	211110_s_at	GPL570	[36,37]
GSE29044	2014	Saudi Arabia	Affymetrix Human Genome U133 Plus 2.0 Array	211110_s_at	GPL570	[38]
GSE37181	2014	Italy	Illumina HumanWG-6 v3.0 expression beadchip	ILMN_1659572	GPL6884	[39]
GSE48390	2014	Taiwan	Affymetrix Human Genome U133 Plus 2.0 Array	211110_s_at	GPL570	[40]
GSE43365	2013	USA	Affymetrix Human Genome U133 Plus 2.0 Array	211110_s_at	GPL570	[41]
GSE45255	2013	USA	Affymetrix Human Genome U133A Array	211621_at	GPL96	[42]
GSE35629	2013	USA	Agilent Human 1A Oligo UNC custom Microarrays	14806	GPL1390	[43]
GSE29210	2013	Canada	Agilent-014850 Whole Human Genome Microarray 4x44K G4112F	A_23_P113111	GPL6480	[44]
GSE34138	2012	Netherlands	Illumina HumanWG-6 v3.0 expression beadchip	ILMN_1659572	GPL6884	[45,46]

GSE30682	2012	France	Illumina HumanWG-6 v3.0 expression beadchip	ILMN_1659572	GPL6884	[47]
GSE10893	2011	USA	Agilent-011521 Human 1A Microarray G4110A	14806	GPL885	[48]
GSE16987	2011	Canada	Illumina humanRef-8 v2.0 expression beadchip	ILMN_1659572	GPL6104	[49,50]
GSE22597	2011	USA	Affymetrix Human Genome U133A Array	211621_at	GPL96	[51]
GSE26639	2011	France	Affymetrix Human Genome U133 Plus 2.0 Array	211110_s_at	GPL570	[52]
GSE26304	2010	Canada	Agilent-012391 Whole Human Genome Oligo Microarray G4112A	A_23_P113111	GPL6848	[53]
GSE20271	2010	USA	Affymetrix Human Genome U133A Array	211621_at	GPL96	[54,55]
GSE20624	2010	USA	Agilent-011521 Human 1A Microarray G4110A	14806	GPL1390	[56]
GSE21653	2010	France	Affymetrix Human Genome U133 Plus 2.0 Array	211110_s_at	GPL570	[57,58]
GSE10510	2008	Germany	DKFZ Division of Molecular Genome Analysis Human Operon 4.0 oligo Array 35k	H300021349	GPL6486	[59]
GSE1561	2005	France	Affymetrix Human Genome U133A Array	211621_at	GPL96	[60]
GSE2034	2005	USA	Affymetrix Human Genome U133A Array	211621_at	GPL96	[61]
GSE17907	2009	France	Affymetrix Human Genome U133 Plus 2.0 Array	211110_s_at	GPL570	[62]
GSE19615	2010	USA	Affymetrix Human Genome U133 Plus 2.0 Array	211110_s_at	GPL570	[63]
GSE126870	2020	United Kingdom	Illumina HumanHT-12 V4.0 expression beadchip	ILMN_1767351	GPL10558	[64]
GSE76727	2016	United Kingdom	Illumina HumanHT-12 V4.0 expression beadchip	ILMN_1767351	GPL10558	[65]
GSE55639	2014	Switzerland	Illumina HumanHT-12 V4.0 expression beadchip	ILMN_1767351	GPL10558	[66]
GSE78958	2016	USA	Affymetrix Human Genome U133A 2.0 Array	211110_s_at	GPL571	[67]
GSE11121	2008	Germany	Affymetrix Human Genome U133A Array	211621_at	GPL96	[68–71]

GSE88847	2019	Canada	Affymetrix Human Gene 1.0 ST Array	367_at	GPL20171	[72]
GSE4056	2006	Germany	DKFZ/Operon Human Oligo Set v2.1	10496	GPL3367	[73]
GSE102484	2017	Taiwan	Affymetrix Human Genome U133 Plus 2.0 Array	211110_s_at	GPL570	[74]
GSE19697	2009	USA	Affymetrix Human Genome U133 Plus 2.0 Array	211110_s_at	GPL570	[75]
GSE58812	2015	France	Affymetrix Human Genome U133 Plus 2.0 Array	211110_s_at	GPL570	[76]
GSE104549	2018	Italy	Illumina HumanWG-6 v3.0 expression beadchip	ILMN_1659572	GPL6884	[77]
GSE45827	2016	France	Affymetrix Human Genome U133 Plus 2.0 Array	211110_s_at	GPL570	[78]

References

1. Aure MR, Vitelli V, Jernström S, Kumar S, Krohn M, Due EU, et al. Integrative clustering reveals a novel split in the luminal A subtype of breast cancer with impact on outcome. *Breast Cancer Res*. **2017**;19(1):44. doi: 10.1186/s13058-017-0812-y.
2. Knutsen E, Lellahi SM, Aure MR, Nord S, Fismen S, Larsen KB, et al. The expression of the long NEAT1_2 isoform is associated with human epidermal growth factor receptor 2-positive breast cancers. *Sci Rep*. **2020** Jan 28;10(1):1277. doi: 10.1038/s41598-020-57759-4.
3. Pladsen A V, Nilsen G, Rueda OM, Aure MR, Borgan Ø, Liestøl K, et al. DNA copy number motifs are strong and independent predictors of survival in breast cancer. *Commun Biol*. **2020**;3(1):153. doi: 10.1038/s42003-020-0884-6.
4. Malvia S, Bagadi SAR, Pradhan D, Chintamani C, Bhatnagar A, Arora D, et al. Study of Gene Expression Profiles of Breast Cancers in Indian Women. *Sci Rep*. **2019**;9(1):10018. doi: 10.1038/s41598-019-46261-1.
5. Varadan V, Gilmore H, Miskimen KLS, Tuck D, Parsai S, Awadallah A, et al. Immune Signatures Following Single Dose Trastuzumab Predict Pathologic Response to PreoperativeTrastuzumab and Chemotherapy in HER2-Positive Early Breast Cancer. *Clin Cancer Res*. **2016**;22(13):3249–59. doi: 10.1158/1078-0432.CCR-15-2021
6. Larsen MJ, Thomassen M, Tan Q, Lænkholm A-V, Bak M, Sørensen KP, et al. RNA profiling reveals familial aggregation of molecular subtypes in non-BRCA1/2 breast cancer families. *BMC Med Genomics*. **2014** Jan 31;7:9. doi: 10.1186/1755-8794-7-9.
7. Huang X, Dugo M, Callari M, Sandri M, De Cecco L, Valeri B, et al. Molecular portrait of breast cancer in China reveals comprehensive transcriptomic likeness to Caucasian breast cancer and low prevalence of luminal A subtype. *Cancer Med*. **2015** Jul;4(7):1016–30. doi: 10.1002/cam4.442.
8. Maubant S, Tesson B, Maire V, Ye M, Rigaill G, Gentien D, et al. Transcriptome analysis of Wnt3a-treated triple-negative breast cancer cells. *PLoS One*. **2015**;10(4):e0122333. doi: 10.1371/journal.pone.0122333
9. Maire V, Baldeyron C, Richardson M, Tesson B, Vincent-Salomon A, Gravier E, et al. TTK/hMPS1 is an attractive therapeutic target for triple-negative breast cancer. *PLoS One*. **2013**;8(5):e63712. doi: 10.1371/journal.pone.0063712.
10. Maire V, Némati F, Richardson M, Vincent-Salomon A, Tesson B, Rigaill G, et al. Polo-like kinase 1: a potential therapeutic option in combination with

conventional chemotherapy for the management of patients with triple-negative breast cancer. *Cancer Res*. **2013** Jan 15;73(2):813–23. doi: 10.1158/0008-5472.CAN-12-2633.
11. Saal LH, Vallon-Christersson J, Häkkinen J, Hegardt C, Grabau D, Winter C, et al. The Sweden Cancerome Analysis Network - Breast (SCAN-B) Initiative: a large-scale multicenter infrastructure towards implementation of breast cancer genomic analyses in the clinical routine. *Genome Med*. **2015**;7(1):20. doi: 10.1186/s13073-015-0131-9.
12. Clarke C, Madden SF, Doolan P, Aherne ST, Joyce H, O'Driscoll L, et al. Correlating transcriptional networks to breast cancer survival: a large-scale coexpression analysis. *Carcinogenesis*. **2013** Oct;34(10):2300–8. doi: 10.1093/carcin/bgt208.
13. Carraro DM, Koike Folgueira MAA, Garcia Lisboa BC, Ribeiro Olivieri EH, Vitorino Krepischi AC, de Carvalho AF, et al. Comprehensive analysis of BRCA1, BRCA2 and TP53 germline mutation and tumor characterization: a portrait of early-onset breast cancer in Brazil. *PLoS One*. **2013**;8(3):e57581. doi: 10.1371/journal.pone.0057581.
14. Bastos EP, Brentani H, Pasini FS, Silva ART, Torres CH, Puga RD, et al. MicroRNAs discriminate familial from sporadic non-BRCA1/2 breast carcinoma arising in patients ≤35 years. *PLoS One*. **2014**;9(7):e101656. doi: 10.1371/journal.pone.0101656.
15. Horak CE, Pusztai L, Xing G, Trifan OC, Saura C, Tseng L-M, et al. Biomarker analysis of neoadjuvant doxorubicin/cyclophosphamide followed by ixabepilone or Paclitaxel in early-stage breast cancer. *Clin Cancer Res*. **2013** Mar 15;19(6):1587–95. doi: 10.1158/1078-0432.CCR-12-1359.
16. Jönsson G, Staaf J, Vallon-Christersson J, Ringnér M, Gruvberger-Saal SK, Saal LH, et al. The retinoblastoma gene undergoes rearrangements in BRCA1-deficient basal-like breast cancer. *Cancer Res*. **2012** Aug 15;72(16):4028–36. doi: 10.1158/0008-5472.CAN-12-0097.
17. Molloy TJ, Roepman P, Naume B, van't Veer LJ. A prognostic gene expression profile that predicts circulating tumor cell presence in breast cancer patients. *PLoS One*. **2012**;7(2):e32426. doi: 10.1371/journal.pone.0032426.
18. Dedeurwaerder S, Desmedt C, Calonne E, Singhal SK, Haibe-Kains B, Defrance M, et al. DNA methylation profiling reveals a predominant immune component in breast cancers. *EMBO Mol Med*. **2011** Dec;3(12):726–41. doi: 10.1002/emmm.201100801.
19. Niméus-Malmström E, Krogh M, Malmström P, Strand C, Fredriksson I, Karlsson P, et al. Gene expression profiling in primary breast cancer distinguishes patients developing local recurrence after breast-conservation surgery, with or without postoperative radiotherapy. *Breast Cancer Res*. **2008**;10(2):R34. doi: 10.1186/bcr1997.
20. Creighton CJ, Sada YH, Zhang Y, Tsimelzon A, Wong H, Dave B, et al. A gene transcription signature of obesity in breast cancer. *Breast Cancer Res Treat*. **2012** Apr;132(3):993–1000. doi: 10.1007/s10549-011-1595-y.
21. Buffa FM, Camps C, Winchester L, Snell CE, Gee HE, Sheldon H, et al. microRNA-associated progression pathways and potential therapeutic targets identified by integrated mRNA and microRNA expression profiling in breast cancer. *Cancer Res*. **2011** Sep 1;71(17):5635–45. doi: 10.1158/0008-5472.CAN-11-0489.
22. Popovici V, Chen W, Gallas BG, Hatzis C, Shi W, Samuelson FW, et al. Effect of training-sample size and classification difficulty on the accuracy of genomic predictors. *Breast Cancer Res*. **2010**;12(1):R5. doi: 10.1186/bcr2468.
23. Shi L, Campbell G, Jones WD, Campagne F, Wen Z, Walker SJ, et al. The MicroArray Quality Control (MAQC)-II study of common practices for the development and validation of microarray-based predictive models. *Nat Biotechnol*. **2010** Aug;28(8):827–38. doi: 10.1038/nbt.1665.
24. Pedraza V, Gomez-Capilla JA, Escaramis G, Gomez C, Torné P, Rivera JM, et al. Gene expression signatures in breast cancer distinguish phenotype characteristics, histologic subtypes, and tumor invasiveness. *Cancer*. **2010** Jan 15;116(2):486–96. doi: 10.1002/cncr.24805.
25. Chin SF, Teschendorff AE, Marioni JC, Wang Y, Barbosa-Morais NL, Thorne NP, et al. High-resolution aCGH and expression profiling identifies a novel genomic subtype of ER negative breast cancer. *Genome Biol*. **2007**;8(10):R215. doi: 10.1186/gb-2007-8-10-r215.
26. Zhang Y, Martens JWM, Yu JX, Jiang J, Sieuwerts AM, Smid M, et al. Copy number alterations that predict metastatic capability of human breast cancer. *Cancer Res*. **2009** May 1;69(9):3795–801. doi: 10.1158/0008-5472.CAN-08-4596.
27. Desmedt C, Piette F, Loi S, Wang Y, Lallemand F, Haibe-Kains B, et al. Strong time dependence of the 76-gene prognostic signature for node-negative breast cancer patients in the TRANSBIG multicenter independent validation series. *Clin Cancer Res*. **2007** Jun 1;13(11):3207–14. doi: 10.1158/1078-0432.CCR-06-2765.

28. Patil P, Bachant-Winner P-O, Haibe-Kains B, Leek JT. Test set bias affects reproducibility of gene signatures. *Bioinformatics*. **2015** Jul 15;31(14):2318–23. doi: 10.1093/bioinformatics/btv157.
29. Sinn B V, Fu C, Lau R, Litton J, Tsai T-H, Murthy R, et al. SETER/PR: a robust 18-gene predictor for sensitivity to endocrine therapy for metastatic breast cancer. *NPJ breast cancer*. **2019**;5:16. doi: 10.1038/s41523-019-0111-0.
30. Zoeller JJ, F. Press M, Selfors LM, Dering J, Slamon DJ, Hurvitz SA, et al. Clinical evaluation of BCL-2/XL levels pre- and post- HER2-targeted therapy. *PLoS One*. **2021**;1–17. doi: 10.1371/journal.pone.0251163.
31. Sjöström M, Staaf J, Edén P, Wärnberg F, Bergh J, Malmström P, et al. Identification and validation of single-sample breast cancer radiosensitivity gene expression predictors. *Breast Cancer Res*. **2018**;20(1):64. doi: 10.1186/s13058-018-0978-y.
32. Prabhakaran S, Rizk VT, Ma Z, Cheng C-H, Berglund AE, Coppola D, et al. Evaluation of invasive breast cancer samples using a 12-chemokine gene expression score: correlation with clinical outcomes. *Breast Cancer Res*. **2017**;19(1):71. doi: 10.1186/s13058-017-0864-z.
33. Wang J, Zhang X, Beck AH, Collins LC, Chen WY, Tamimi RM, et al. Alcohol Consumption and Risk of Breast Cancer by Tumor Receptor Expression. *Horm Cancer*. **2015** Dec;6(5–6):237–46. doi: 10.1007/s12672-015-0235-0.
34. Lesurf R, Aure MR, Mørk HH, Vitelli V, Oslo Breast Cancer Research Consortium (OSBREAC), Lundgren S, et al. Molecular Features of Subtype-Specific Progression from Ductal Carcinoma In Situ to Invasive Breast Cancer. *Cell Rep*. **2016**;16(4):1166–79. doi: 10.1016/j.celrep.2016.06.051.
35. Xiang J, Hurchla MA, Fontana F, Su X, Amend SR, Esser AK, et al. CXCR4 Protein Epitope Mimetic Antagonist POL5551 Disrupts Metastasis and Enhances Chemotherapy Effect in Triple-Negative Breast Cancer. *Mol Cancer Ther*. **2015** Nov;14(11):2473–85. doi: 10.1158/1535-7163.MCT-15-0252.
36. Grinchuk O V, Motakis E, Yenamandra SP, Ow GS, Jenjaroenpun P, Tang Z, et al. Sense-antisense gene-pairs in breast cancer and associated pathological pathways. *Oncotarget*. **2015** Dec 8;6(39):42197–221. doi: 10.18632/oncotarget.6255.
37. Aswad L, Yenamandra SP, Ow GS, Grinchuk O, Ivshina A V, Kuznetsov VA. Genome and transcriptome delineation of two major oncogenic pathways governing invasive ductal breast cancer development. *Oncotarget*. **2015** Nov 3;6(34):36652–74. doi: 10.18632/oncotarget.5543.
38. Colak D, Nofal A, Albakheet A, Nirmal M, Jeprel H, Eldali A, et al. Age-specific gene expression signatures for breast tumors and cross-species conserved potential cancer progression markers in young women. *PLoS One*. **2013**;8(5):e63204. doi: 10.1371/journal.pone.0063204.
39. Callari M, Musella V, Di Buduo E, Sensi M, Miodini P, Dugo M, et al. Subtype-dependent prognostic relevance of an interferon-induced pathway metagene in node-negative breast cancer. *Mol Oncol*. **2014** Oct;8(7):1278–89. doi: 10.1016/j.molonc.2014.04.010.
40. Huang C-C, Tu S-H, Lien H-H, Jeng J-Y, Huang C-S, Huang C-J, et al. Concurrent gene signatures for han chinese breast cancers. *PLoS One*. **2013**;8(10):e76421. doi: 10.1371/journal.pone.0076421.
41. Metzger-Filho O, Catteau A, Michiels S, Buyse M, Ignatiadis M, Saini KS, et al. Genomic Grade Index (GGI): feasibility in routine practice and impact on treatment decisions in early breast cancer. *PLoS One*. **2013**;8(8):e66848. doi: 10.1371/journal.pone.0066848.
42. Nagalla S, Chou JW, Willingham MC, Ruiz J, Vaughn JP, Dubey P, et al. Interactions between immunity, proliferation and molecular subtype in breast cancer prognosis. *Genome Biol*. **2013** Apr 29;14(4):R34. doi: 10.1186/gb-2013-14-4-r34.
43. Ulirsch J, Fan C, Knafl G, Wu MJ, Coleman B, Perou CM, et al. Vimentin DNA methylation predicts survival in breast cancer. *Breast Cancer Res Treat*. **2013** Jan;137(2):383–96. doi: 10.1007/s10549-012-2353-5.
44. Bastani M, Vos L, Asgarian N, Deschenes J, Graham K, Mackey J, et al. A machine learned classifier that uses gene expression data to accurately predict estrogen receptor status. *PLoS One*. **2013**;8(12):e82144. doi: 10.1371/journal.pone.0082144.
45. de Ronde JJ, Lips EH, Mulder L, Vincent AD, Wesseling J, Nieuwland M, et al. SERPINA6, BEX1, AGTR1, SLC26A3, and LAPTM4B are markers of resistance to neoadjuvant chemotherapy in HER2-negative breast cancer. *Breast Cancer Res Treat*. **2013** Jan;137(1):213–23. doi: 10.1007/s10549-012-2340-x.
46. Kersten K, Coffelt SB, Hoogstraat M, Verstegen NJM, Vrijland K, Ciampricotti M, et al. Mammary tumor-derived CCL2 enhances pro-metastatic systemic inflammation through upregulation of IL1β in tumor-associated macrophages. *Oncoimmunology*. 6(8):e1334744. doi: 10.1080/2162402X.2017.1334744.
47. Servant N, Bollet MA, Halfwerk H, Bleakley K, Kreike B, Jacob L, et al. Search for a gene expression signature of breast cancer local recurrence in young women.

Clin Cancer Res. **2012** Mar 15;18(6):1704–15. doi: 10.1158/1078-0432.CCR-11-1954.
48. Weigman VJ, Chao H-H, Shabalin AA, He X, Parker JS, Nordgard SH, et al. Basal-like Breast cancer DNA copy number losses identify genes involved in genomic instability, response to therapy, and patient survival. *Breast Cancer Res Treat*. **2012** Jun;133(3):865–80. doi: 10.1007/s10549-011-1846-y.
49. Wang D-Y, Done SJ, McCready DR, Boerner S, Kulkarni S, Leong WL. A new gene expression signature, the ClinicoMolecular Triad Classification, may improve prediction and prognostication of breast cancer at the time of diagnosis. *Breast Cancer Res*. **2011** Sep 22;13(5):R92. doi: 10.1186/bcr3017.
50. Wang D-Y, Done SJ, Mc Cready DR, Leong WL. Validation of the prognostic gene portfolio, ClinicoMolecular Triad Classification, using an independent prospective breast cancer cohort and external patient populations. *Breast Cancer Res*. **2014** Jul 4;16(4):R71. doi: 10.1186/bcr3686.
51. Iwamoto T, Bianchini G, Qi Y, Cristofanilli M, Lucci A, Woodward WA, et al. Different gene expressions are associated with the different molecular subtypes of inflammatory breast cancer. *Breast Cancer Res Treat*. **2011** Feb;125(3):785–95. doi: 10.1007/s10549-010-1280-6.
52. de Cremoux P, Valet F, Gentien D, Lehmann-Che J, Scott V, Tran-Perennou C, et al. Importance of pre-analytical steps for transcriptome and RT-qPCR analyses in the context of the phase II randomised multicentre trial REMAGUS02 of neoadjuvant chemotherapy in breast cancer patients. *BMC Cancer*. **2011** Jun 1;11:215. doi: 10.1186/1471-2407-11-215.
53. Muggerud AA, Hallett M, Johnsen H, Kleivi K, Zhou W, Tahmasebpoor S, et al. Molecular diversity in ductal carcinoma in situ (DCIS) and early invasive breast cancer. *Mol Oncol*. **2010** Aug;4(4):357–68. doi: 10.1016/j.molonc.2010.06.007.
54. Tabchy A, Valero V, Vidaurre T, Lluch A, Gomez H, Martin M, et al. Evaluation of a 30-gene paclitaxel, fluorouracil, doxorubicin, and cyclophosphamide chemotherapy response predictor in a multicenter randomized trial in breast cancer. *Clin Cancer Res*. **2010** Nov 1;16(21):5351–61. doi: 10.1158/1078-0432.CCR-10-1265.
55. Shen K, Song N, Kim Y, Tian C, Rice SD, Gabrin MJ, et al. A systematic evaluation of multi-gene predictors for the pathological response of breast cancer patients to chemotherapy. *PLoS One*. **2012**;7(11):e49529. doi: 10.1371/journal.pone.0049529.
56. Anders CK, Fan C, Parker JS, Carey LA, Blackwell KL, Klauber-DeMore N, et al. Breast carcinomas arising at a young age: unique biology or a surrogate for aggressive intrinsic subtypes? *J Clin Oncol*. **2011** Jan 1;29(1):e18-20. doi: 10.1200/JCO.2010.28.9199.
57. Sabatier R, Finetti P, Cervera N, Lambaudie E, Esterni B, Mamessier E, et al. A gene expression signature identifies two prognostic subgroups of basal breast cancer. *Breast Cancer Res Treat*. **2011** Apr;126(2):407–20. doi: 10.1007/s10549-010-0897-9.
58. Sabatier R, Finetti P, Adelaide J, Guille A, Borg J-P, Chaffanet M, et al. Down-regulation of ECRG4, a candidate tumor suppressor gene, in human breast cancer. *PLoS One*. **2011**;6(11):e27656. Available from: http://www.ncbi.nlm.nih.gov/pubmed/22110708
59. Calabrò A, Beissbarth T, Kuner R, Stojanov M, Benner A, Asslaber M, et al. Effects of infiltrating lymphocytes and estrogen receptor on gene expression and prognosis in breast cancer. *Breast Cancer Res Treat*. **2009** Jul;116(1):69–77. doi: 10.1007/s10549-008-0105-3.
60. Farmer P, Bonnefoi H, Becette V, Tubiana-Hulin M, Fumoleau P, Larsimont D, et al. Identification of molecular apocrine breast tumours by microarray analysis. *Oncogene*. **2005** Jul 7;24(29):4660–71. doi: 10.1038/sj.onc.1208561.
61. Wang Y, Klijn JGM, Zhang Y, Sieuwerts AM, Look MP, Yang F, et al. Gene-expression profiles to predict distant metastasis of lymph-node-negative primary breast cancer. *Lancet (London, England)*. 365(9460):671–9. doi: 10.1016/S0140-6736(05)17947-1.
62. Sircoulomb F, Bekhouche I, Finetti P, Adélaïde J, Ben Hamida A, Bonansea J, et al. Genome profiling of ERBB2-amplified breast cancers. *BMC Cancer*. **2010** Oct 8;10:539. doi: 10.1186/1471-2407-10-539.
63. Li Y, Zou L, Li Q, Haibe-Kains B, Tian R, Li Y, et al. Amplification of LAPTM4B and YWHAZ contributes to chemotherapy resistance and recurrence of breast cancer. *Nat Med*. **2010** Feb;16(2):214–8. doi: 10.1038/nm.2090.
64. Gao Q, López-Knowles E, Cheang MCU, Morden J, Ribas R, Sidhu K, et al. Impact of aromatase inhibitor treatment on global gene expression and its association with antiproliferative response in ER+ breast cancer in postmenopausal patients. *Breast Cancer Res*. **2019**;22(1):2. doi: 10.1186/s13058-019-1223-z.
65. Pearce DA, Arthur LM, Turnbull AK, Renshaw L, Sabine VS, Thomas JS, et al. Tumour sampling method can significantly influence gene expression profiles derived from neoadjuvant window studies. *Sci Rep*. **2016**;6:29434. doi: 10.1038/srep29434.

66. Piscuoglio S, Ng CKY, Martelotto LG, Eberle CA, Cowell CF, Natrajan R, et al. Integrative genomic and transcriptomic characterization of papillary carcinomas of the breast. *Mol Oncol*. **2014** Dec;8(8):1588–602. doi: 10.1016/j.molonc.2014.06.011.
67. Toro AL, Costantino NS, Shriver CD, Ellsworth DL, Ellsworth RE. Effect of obesity on molecular characteristics of invasive breast tumors: gene expression analysis in a large cohort of female patients. *BMC Obes*. **2016**;3:22. doi: 10.1186/s40608-016-0103-7.
68. Schmidt M, Böhm D, von Törne C, Steiner E, Puhl A, Pilch H, et al. The humoral immune system has a key prognostic impact in node-negative breast cancer. *Cancer Res*. **2008** Jul 1;68(13):5405–13. doi: 10.1158/0008-5472.CAN-07-5206.
69. Cadenas C, van de Sandt L, Edlund K, Lohr M, Hellwig B, Marchan R, et al. Loss of circadian clock gene expression is associated with tumor progression in breast cancer. *Cell Cycle*. **2014**;13(20):3282–91. doi: 10.4161/15384101.2014.954454.
70. Hellwig B, Hengstler JG, Schmidt M, Gehrmann MC, Schormann W, Rahnenführer J. Comparison of scores for bimodality of gene expression distributions and genome-wide evaluation of the prognostic relevance of high-scoring genes. *BMC Bioinformatics*. **2010** May 25;11:276. doi: 10.1186/1471-2105-11-276.
71. Heimes A-S, Härtner F, Almstedt K, Krajnak S, Lebrecht A, Battista MJ, et al. Prognostic Significance of Interferon-γ and Its Signaling Pathway in Early Breast Cancer Depends on the Molecular Subtypes. *Int J Mol Sci*. **2020** Sep 29;21(19). doi: 10.3390/ijms21197178.
72. Gruosso T, Gigoux M, Manem VSK, Bertos N, Zuo D, Perlitch I, et al. Spatially distinct tumor immune microenvironments stratify triple-negative breast cancers. *J Clin Invest*. **2019**;129(4):1785–800. doi: 10.1172/JCI96313.
73. Thuerigen O, Schneeweiss A, Toedt G, Warnat P, Hahn M, Kramer H, et al. Gene expression signature predicting pathologic complete response with gemcitabine, epirubicin, and docetaxel in primary breast cancer. *J Clin Oncol*. **2006** Apr 20;24(12):1839–45. doi: 10.1200/JCO.2005.04.7019.
74. Cheng SH-C, Huang T-T, Cheng Y-H, Tan TBK, Horng C-F, Wang YA, et al. Validation of the 18-gene classifier as a prognostic biomarker of distant metastasis in breast cancer. *PLoS One*. **2017**;12(9):e0184372. doi: 10.1371/journal.pone.0184372.
75. Lin Y, Lin S, Watson M, Trinkaus KM, Kuo S, Naughton MJ, et al. A gene expression signature that predicts the therapeutic response of the basal-like breast cancer to neoadjuvant chemotherapy. *Breast Cancer Res Treat*. **2010** Oct;123(3):691–9. doi: 10.1007/s10549-009-0664-y.
76. Jézéquel P, Loussouarn D, Guérin-Charbonnel C, Campion L, Vanier A, Gouraud W, et al. Gene-expression molecular subtyping of triple-negative breast cancer tumours: importance of immune response. *Breast Cancer Res*. **2015** Mar 20;17:43. doi: 10.1186/s13058-015-0550-y.
77. Cappelletti V, Iorio E, Miodini P, Silvestri M, Dugo M, Daidone MG. Metabolic Footprints and Molecular Subtypes in Breast Cancer. *Dis Markers*. **2017**;2017:7687851. doi: 10.1155/2017/7687851.
78. Gruosso T, Mieulet V, Cardon M, Bourachot B, Kieffer Y, Devun F, et al. Chronic oxidative stress promotes H2AX protein degradation and enhances chemosensitivity in breast cancer patients. *EMBO Mol Med*. **2016**;8(5):527–49. doi: 10.15252/emmm.201505891.

Table S2. Heterogeneity analysis and publication bias based on Egger's test

		After sensitivity analysis							
		**Q-value**	**df (Q)**	**P-value**	**I-squared**	**Egger regression intercept**	**95% lower limit**	**95% upper limit**	**p-value**
**Molecular subtypes**	Basal-like Vs Luminal A	7.348	6	0.289	18.346	1.360	-4.993	7.714	0.619
	Basal-like Vs Luminal B	5.736	6	0.453	0	0.815	-3.671	5.302	0.686
	Basal-like Vs HER2-enriched	39.449	9	< 0.001	77.186	-1.107	-5.575	3.330	0.580
	Basal-like Vs Normal-like	13.336	6	0.037	55.010	0.181	-4.606	4.969	0.926
	Normal-like Vs Luminal A	18.555	12	0.099	35.329	0.327	-2.099	2.755	0.771
	Normal-like Vs Luminal B	14.0618	13	0.369	7.551	0.810	-0.798	2.420	0.293
	Normal-like Vs HER2-enriched	9.982	9	0.351	9.839	0.696	-1.175	2.567	0.415
	HER2-enriched Vs Luminal A	30.872	14	0.005	54.651	1.168	-1.298	3.635	0.324
	HER2-enriched Vs Luminal B	17.168	10	0.070	41.753	0.038	-2.301	2.378	0.971
	Luminal B Vs Luminal A	15.318	10	0.120	34.721	-0.909	-2.905	1.087	0.329
**IHC-surrogate subtypes**	TNBC Vs Luminal A	12.252	7	0.092	42.867	2.886	-0.143	5.915	0.058
	TNBC Vs Luminal B	19.594	5	0.001	74.482	2.420	-8.412	13.253	0.590
	TNBC Vs HER2-enriched	8.841	6	0.182	32.141	-0.680	-6.237	4.876	0.765
	HER2-enriched Vs Luminal A	14.084	10	0.169	29.000	1.376	-2.170	4.922	0.402
	HER2-enriched Vs Luminal B	11.043	10	0.354	9.445	-0.657	-4.503	3.189	0.708
	Luminal B vs Luminal A	5.868	8	0.662	0	-1.417	-4.276	1.440	0.279

		Primary analysis							
		**Q-value**	**df (Q)**	**P-value**	**I-squared**	**Egger regression intercept**	**95% lower limit**	**95% upper limit**	**p-value**
**Molecular subtypes**	Basal-like Vs Luminal A	532.929	14	< 0.001	97.373	7.002	-0.086	14.091	0.052
	Basal-like Vs Luminal B	219.493	14	< 0.001	93.621	5.699	1.843	9.555	0.005
	Basal-like Vs HER2-enriched	190.020	14	< 0.001	92.632	3.859	-0.189	7.908	0.061
	Basal-like Vs Normal-like	158.571	10	< 0.001	93.693	5.222	2.160	8.284	0.002
	Normal-like Vs Luminal A	67.882	14	< 0.001	79.376	0.169	-3.154	3.493	0.916
	Normal-like Vs Luminal B	26.253	14	0.024	46.673	1.716	-0.163	3.596	0.071
	Normal-like Vs HER2-enriched	17.984	10	0.055	44.397	0.999	-0.689	2.689	0.226
	HER2-enriched Vs Luminal A	30.872	14	0.005	54.651	0.934	-1.114	2.984	0.351
	HER2-enriched Vs Luminal B	17.168	10	0.070	41.753	-0.417	-2.555	1.720	0.683
	Luminal B Vs Luminal A	15.318	10	0.120	34.721	-1.386	-2.982	0.210	0.083
**IHC-surrogate subtypes**	TNBC Vs Luminal A	228.772	12	< 0.001	94.754	6.330	0.657	12.003	0.031
	TNBC Vs Luminal B	131.666	9	< 0.001	93.164	4.212	-2.529	10.953	0.200
	TNBC Vs HER2-enriched	76.255	10	< 0.001	86.886	4.243	-3.227	11.713	0.241
	HER2-enriched Vs Luminal A	14.084	10	0.169	29.000	1.835	-1.195	4.865	0.215
	HER2-enriched Vs Luminal B	11.043	10	0.354	9.445	3.147	-1.387	7.683	0.157
	Luminal B vs Luminal A	5.868	8	0.662	0	-0.815	-3.780	2.108	0.548
